# FTIR Characterization and Bioactivity Assessment of *Cinnamomum camphora* Essential Oil: Antioxidant, Anti‐Enzymatic, and Antifungal Properties Against Phytopathogens

**DOI:** 10.1002/cbdv.202500720

**Published:** 2025-06-16

**Authors:** Maroua Cheribot Cherif, Hicham Boughendjioua, Lucia Caputo, Ippolito Camele, Tarek Tahraoui, Vincenzo De Feo, Hazem S. Elshafie

**Affiliations:** ^1^ Laboratory of Physical Chemistry and Biology of Materials Higher Normal School of Technological Education of Skikda Skikda Algeria; ^2^ Department of Pharmacy University of Salerno Fisciano Italy; ^3^ Department of Agricultural, Forestry, Food and Environmental Sciences University of Basilicata Potenza Italy; ^4^ Mines Metallurgy Materials Laboratory L3M National Higher School of Technology and Engineering‐ENSTI Annaba Annaba Algeria

**Keywords:** antioxidant activity, anti‐enzymatic activities, antifungal activities, *Cinnamomum camphora*, essential oil, FTIR analysis

## Abstract

This study explores key biopharmaceutical properties of *Cinnamomum camphora* essential oil (EO), including antioxidant, anti‐enzymatic, and antifungal activities, and evaluates its minimum inhibitory concentration (MIC) against some common phytopathogens. The functional groups of the EO were identified using Fourier‐transform infrared (FTIR) spectroscopy. The antioxidant capacity, evaluated using 2,2‐diphenyl‐1‐picrylhydrazyl (DPPH), ABTS, and ferric‐reducing antioxidant power (FRAP) methods, revealed a moderate activity. The antifungal activity assay showed a complete growth inhibition of *Monilinia laxa*, *Monilinia fructicola*, *Sclerotinia sclerotiorum*, *Colletotrichum gloeosporioides*, and *Botrytis cinerea* at a concentration of 10 000 ppm of the tested EO. However, the lowest antifungal activity (17.8%) was observed against *Aspergillus niger* only at the highest tested concentration. The measured MIC was 7000 ppm for *M*. *laxa* and 8000 ppm for *C*. *gloeosporioides*. The anti‐enzymatic activities of the studied EO demonstrated a low inhibitory effect on cholinesterases (AChE and BChE), but the EO exhibited inhibitory concentration (IC_50_) values at 7.8 ± 0.6 and 2.78 ± 0.7 mg/mL for α‐amylase and α‐glucosidase, indicating a promising antidiabetic effect. This research revealed that the studied EO possesses various biological properties, indicating its potential applicability in several agro‐pharmaceutical fields.

## Graphical abstract

Essential oil from *Cinnamomum camphora* leaves: Chemical profiling using FTIR analysis and bioactivity assessment for diverse bio‐pharmaceutical applications.

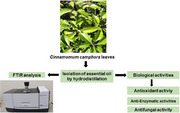

## 1 Introduction

Essential oils (EOs), complex mixtures of volatile secondary metabolites, play a vital role in plant defense mechanisms, ecological interactions, and aromatic properties [1, 2]. Their chemical diversity, derived from plant organs such as leaves, bark, and flowers, is heavily influenced by extraction methods, which, in turn, dictate their biological efficacy. The choice of an extraction method represents a crucial step because it directly influences the chemical composition of EOs and therefore their biological activities [3]. Recent studies highlight EOs as promising therapeutic agents, demonstrating broad‐spectrum pharmacological activities [4, 5, 6].

*Cinnamomum camphora* (L.) J. Presl, commonly known as the camphor tree, is a member of the Lauraceae family, renowned for its industrial and medicinal applications, and has been widely cultivated as ornamental trees and as a source of camphor and camphor oil [7, 8, 9]. Traditionally used in Asian herbal medicine to treat inflammation and rheumatism, its EOs exhibit potential in pharmaceuticals, agriculture, and cosmetics. Various parts of the camphor tree have been used to prepare EOs, which can be used in different fields, such as industry, cosmetics, pesticides, and pharmaceuticals [10]. However, the full scope of its bioactivity—particularly against phytopathogens—remains underexplored.

Camphor EO plays a vital role in combating plant pathogens due to its strong antimicrobial and antifungal properties. It contains bioactive compounds such as camphor, cineole, and borneol, which exhibit broad‐spectrum efficacy against a variety of phytopathogenic microorganisms [11]. Camphor EO has shown promising efficacy in controlling soil‐borne diseases and fungal infections in crops, serving as a natural and friendly alternative to synthetic pesticides. Supporting this, Sobhy et al. [12] investigated the antifungal activity of *C. camphora* methanolic extract against several common phytopathogenic fungi. Their findings demonstrated significant inhibition of mycelial growth in *Fusarium oxysporum*, *Alternaria alternata*, and *Fusarium solani*, with suppression rates of 60%, 49%, and 24%, respectively [12].

Many pathogens, such as bacteria, nematodes, and fungi, can cause plant diseases. Fungal infections of plants are generally the most destructive diseases in agricultural fields [13, 14]. Phytopathogenic fungi pose severe threats to global agriculture, exacerbated by the overuse of synthetic fungicides. While effective, these chemicals contribute to environmental persistence, fungal resistance, and human health risks. Chemical fungicides are widely used in agriculture to protect plants from fungal infections through various mechanisms, including the destruction and inhibition of fungal cells and spores. Moreover, the low cost and simple use of this type of product lead to their overuse [15]. While effective, these chemicals contribute to environmental persistence, fungal resistance, and human health risks due to their low biodegradability and accumulation in the environment [16, 17].

On the other hand, these chemical substances can contaminate soil, water, and air, leading to long‐term ecological damage. Non‐target organisms, including beneficial insects, birds, and aquatic life, may suffer from pesticide exposure, disrupting ecosystems and biodiversity. In humans, prolonged or high‐level exposure to certain pesticides has been linked to respiratory problems, skin disorders, hormonal imbalances, neurological issues, and even cancer. The widespread of chemical pesticides also contributes to the development of resistant pest strains, necessitating the use of even more toxic substances, thereby perpetuating a harmful cycle. This makes the search for new natural alternatives, such as plant‐derived EOs that offer a sustainable alternative due to their biodegradability, low toxicity, and multifunctional properties, including antifungal and antioxidant capacities [18, 19, 20] with low toxicity and low environmental impact [21].

Beyond agriculture, EOs are considered safe for both humans and the environment [22] and show therapeutic promise in managing non‐communicable diseases (NCDs) such as neurodegenerative disorders and diabetes [23]. For instance, acetylcholinesterase (AChE) inhibitors from EOs may alleviate Alzheimer's symptoms [24, 25, 26]. However, α‐amylase/α‐glucosidase inhibitors could mitigate diabetes by modulating postprandial hyperglycemia [21]. Natural inhibitors of these two key enzymes could be developed from plants or EOs for the treatment of diabetes by reducing postprandial hyperglycemia [23, 27]. For all these advantages, plant EOs have been used effectively in pharmaceuticals, food preservatives, cosmetics, and perfumes and could replace or strengthen usual synthetic products [28, 29].

This study investigates the antioxidant, antifungal, and anti‐enzymatic activities of *C. camphora* EO, emphasizing its potential as a biopharmaceutical agent and eco‐compatible fungicide. By elucidating its biochemical properties, we aim to contribute to the development of sustainable solutions for agriculture and medicine. In particular, Fourier‐transform infrared (FTIR) spectroscopy was employed to analyze the studied EO and confirm the presence of key functional groups. The antiradical activity of camphor EO was assessed using three standard assays: 2,2‐diphenyl‐1‐picrylhydrazyl (DPPH), ABTS•⁺ radical scavenging, and ferric‐reducing antioxidant power (FRAP). In addition, the anti‐enzymatic potential was determined by evaluating AChE, α‐amylase, and α‐glucosidase inhibition activities. The antifungal efficacy of camphor EO was tested against seven phytopathogenic fungi: *Monilinia laxa*, *Monilinia fructicola*, *Sclerotinia sclerotiorum*, *Colletotrichum gloeosporioides*, *Aspergillus niger*, *Botrytis cinerea*, and *Plectosphaerella cucumerina*.

## 2 Results and Discussion

### 2.1 Yield and Chemical Composition

The EO yield obtained from *C. camphora* provided a rate of approximately 0.95%. A slight decrease in the yield value was observed compared to our previous study conducted by Cheribot Cherif et al. [30], which may be due to the time of harvest between May 2023 and March 2024 [31]. According to our previous study [30], the gas chromatography–mass spectrometry (GC–MS) analysis allowed the identification of 69 components, which represented 99.57% of the total EO components. The major compounds were camphor (36.81%), α‐pinene (9.91%), d‐limonene (8.63%), and camphene (6.99%), accompanied by other constituents at relatively low levels: β‑myrcene (4.80%), eucalyptol (4.58%), and β‐pinene (3.68%). The identified EO compounds are classified into four main classes: monoterpene hydrocarbons (41.11%), oxygenated monoterpenes (49.22%), sesquiterpene hydrocarbons (9%), and oxygenated sesquiterpene (0.07%) [30].

### 2.2 FTIR Analysis

**FIGURE 1:**
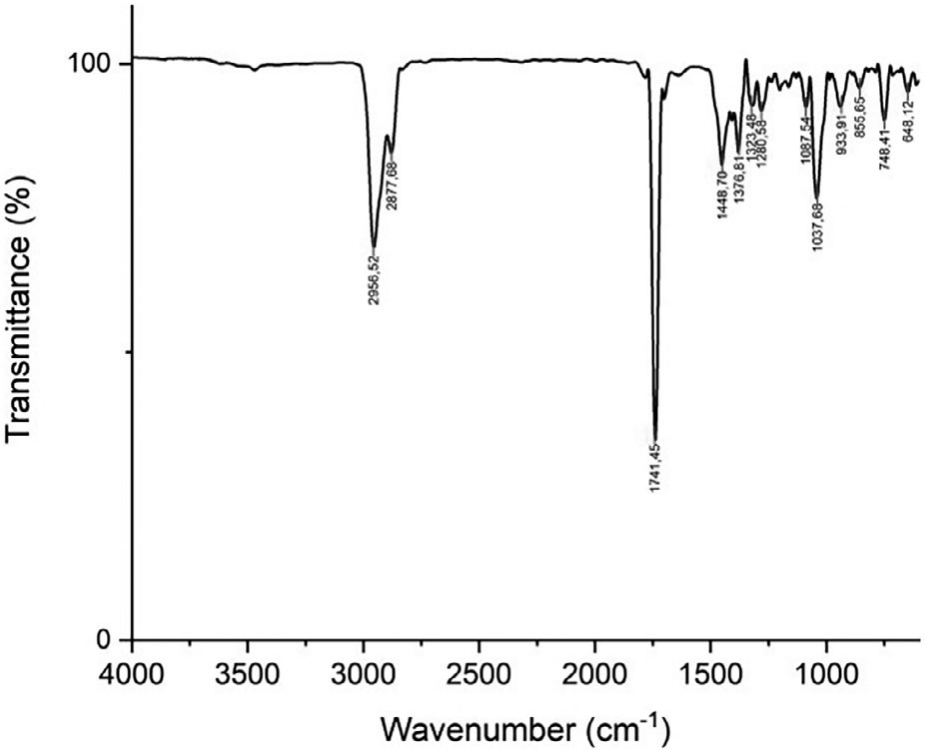
FTIR spectrum of *Cinnamomum camphora* EO.

FTIR spectroscopy was used to identify the functional groups in the studied EO. The results obtained in this study (Figure 1) are in accordance with those previously published by [32, 33], in which the peaks located at around 2956.52, 2877.68, 1741.45, 1448.70, 1376.81, and 748.41 cm^−1^ are attributed to camphor. The spectral bands at 2956.52 and 2877.68 cm^−1^ are due to the asymmetric and symmetric (C–H) stretching in alkanes, respectively [34]. These two bands cause a signal at 1448.70 cm^−1^ [35] that is assigned to CH_3_/CH_2_ bendings [36]. The strong band at 1741.45 cm^−1^ corresponds to the stretching vibration characteristic for the keto groups substituted on a cyclopentane ring system present in camphor [37]. The band at 1376.81 cm^−1^ is due to the deformation vibration of dimethyl‐substituted methylene groups contained in camphor [37]. The peaks at 1323.48 and 1280.56 cm^−1^ could be attributed to the O–H group bending [35]. The band at 1087.54 cm^−1^ is attributed to symmetric stretches of group C–O–C [38]. The spectral band at 1037.68 cm^−1^ corresponds to the C–O deforming in secondary alcohols and aliphatic ethers [39]. The peak at 855.65 cm^−1^ may be linked to the vinylidene C−H out‐of‐plane bend present in camphene [40]. The peak at 748.41 cm^−1^ is assigned to benzene rings CH vibration absorption [41, 42], and the weak band at 648.12 cm^−1^ may correspond to the ring deformation of α‐pinene [36].

### 2.3 Antioxidant Activity

The results obtained showed that our tested EO has a moderate antioxidant activity (Table 1). In DPPH assay, the results showed that the *C. camphora* EO has a low antioxidant power with an IC_50_ value equal to 162.5 µg/mL. This value shows that the studied EO is less effective compared to the standard used (ascorbic acid), whose IC_50_ was 24.3 µg/mL, in agreement with Ioannou et al. [43], who reported that the lower IC_50_ value may indicate the higher antioxidant activity. These obtained results are consistent with those of Kanyal et al. [44], where the measured IC_50_ of the EO from shade‐dried *C. camphora* leaves cultivated in India resulted in 25 ± 0.03 µg/mL. On the other hand, this activity is stronger than that found by Ling et al. [31], who studied the antioxidant activity of the EO extracted from the leaves of seven varieties of *C. camphora* and found that the IC_50_ values ranged between 6.88 ± 0.15 and 28.13 ± 0.44 mg/mL.

**TABLE 1:** Antioxidant activity of *Cinnamomum camphora* essential oil (EO).

	*C. camphora* EO	Ascorbic acid	Trolox
DPPH (IC_50_ µg/mL)	162.5	24.3	—
ABTS (IC_50_ µg/mL)	589.9	—	5.8
FRAP (mmol Fe(II)/g)	12.5	—	5.6

**^Abbreviations:^ :**DPPH, 2,2‐diphenyl‐1‐picrylhydrazyl; FRAP, ferric‐reducing antioxidant power.

In the ABTS assay, the studied EO exhibited low activity, with an IC_50_ of 589.9 µg/mL compared to the standard Trolox, which had an IC_50_ of 5.8 µg/mL. Nevertheless, the *C. camphora* EO demonstrated a higher free radical scavenging ability than those reported by Ling et al. [31], who found IC_50_ values ranging from 19.08 ± 0.02 to 117.22 ±  5.4 mg/mL across seven varieties of *C. camphora* EO. Regarding the FRAP assay, *C. camphora* EO exhibited a value of 12.5 mmol Fe(II)/g, surpassing that of Trolox (5.6 mmol Fe(II)/g). This result aligns with findings by Zakaria et al. [45], who reported that the FRAP value of *C. glanduliferum* leaf EO (63.1 mmol Fe(II)/g) was higher than that observed in ascorbic acid (48.33 mmol Fe(II)/g).

The antioxidant effect of EO is probably related to its chemical profile, especially its monoterpenes. According to a study carried out by Tian et al. [8], the seasonal variation has an effect on monoterpene synthesis in *C. camphora*. The results obtained for the camphor chemotype showed that high temperatures cause an increase in the number of monoterpenes produced and that in summer new monoterpenes appear compared to other seasons (spring and autumn), which makes the choice of the season of use of this plant a crucial step.

### 2.4 Anti‐Enzymatic Activities

The anti‐enzymatic activities were reported in Table 2. Cholinesterase inhibitory activity was evaluated by the ability of the EO to inhibit AChE and BChE activities, whereas antidiabetic activity was evaluated by the ability to inhibit α‐amylase and α‐glucosidase. *C. camphora* EO was more active against α‐glucosidase with an IC_50_ value of 2780 µg/mL compared to other enzymes that showed IC_50_ values ranging from 7800 to 20 200 µg/mL compared to galantamine and acarbose used as positive controls.

**TABLE 2:** Inhibitory effects of the *Cinnamomum camphora* essential oil (EO) on acetylcholinesterase (AChE), BChE, α–amylase, and α‐glucosidase.

Enzymes	*C. camphora* EO (IC_50_ µg/mL)	Galantamine (IC_50_ µg/mL)	Acarbose (IC_50_ µg/mL)
AChE	10 500 ± 400	1.2 ± 0.4	—
BChE	20 200 ± 800	4.7 ± 1.4	—
α‐Amylase	7800 ± 600	—	10.0 ± 5.2
α‐Glucosidase	2780 ± 700	—	960.0 ± 0.47

*Note*: Data are the means ± standard deviation of three experiments.

There are few studies on the anti‐enzymatic activity of *C. camphora* EO. In particular, our results corroborate with study of Bai et al. [46] that showed no significant effect of *C. camphora* EO on the activity of AChE but disagree with Jugreet et al. [47] that reported no inhibition on α‐glucosidase. Instead, Rawat et al. [48] reported that *C. camphora* EO presented a high AChE inhibitory potential (53.61% ± 2.66%) compared with *C. tamala* (46.12% ± 1.52%) at the concentration of 1000 µg/mL; the *C. camphora* EO was characterized by the abundance of oxygenated monoterpenes (70.63%).

Available literature reported data also on other species of *Cinnamomum* genus. Kiran et al. [49] studied the potential of *C. zeylanicum* EO that inhibited the activity of both cholinesterases in a dose‐dependent manner. This species also showed a highly effective inhibition against α‐amylase [50] and α‐glucosidase [51]. Likewise, *Cinnamomum chemungianum* and *Cinnamomum wightii* EOs exhibited a good α‐amylase and α‐glucosidase inhibitory activity [52, 53].

Previous studies have described the ability of some compounds present in EOs, such as 1,8‐cineole, α‐pinene, β‐pinene, camphor, γ‐terpinene, eugenol, and carvacrol, to inhibit cholinesterases [54, 55, 56]. Farag et al. [57] also found that some compounds are more effective than others, such as 1,8‐cineole, which showed better inhibition of AChE than crude camphor EO.

### 2.5 Antifungal Activity

**FIGURE 2:**
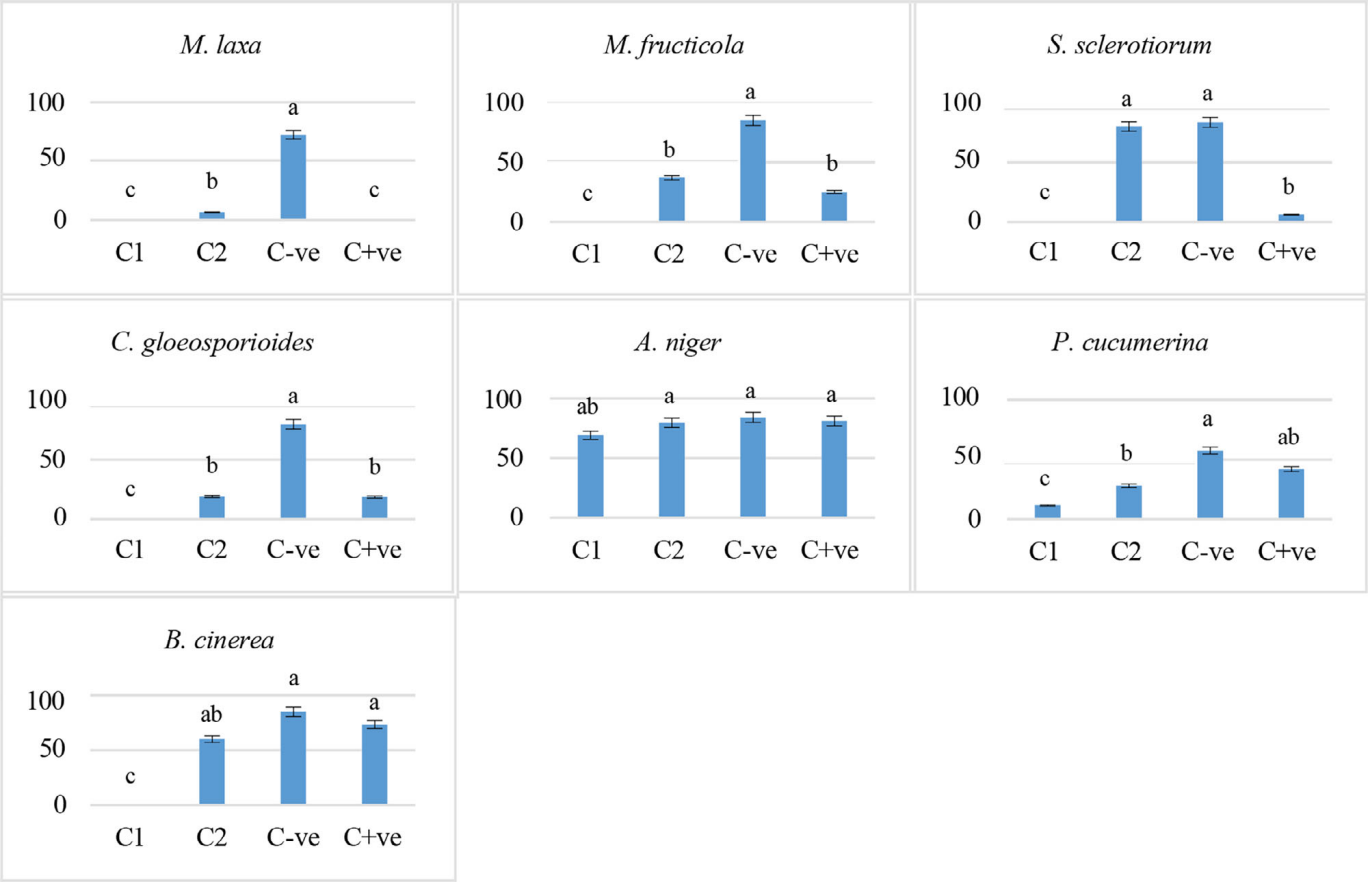
Antifungal activity of the *Cinnamomum camphora* EO expressed as percentage of mycelium growth inhibition (MGI %). C1 is 10 000 ppm, C2 is 7000 ppm, C−ve is potato dextrose agar (PDA), and C+ve is azoxystrobin + cycloheximide. Bars with different letters are significantly different at *p *< 0.05 using the Tukey B test. Data for each bar are expressed as the mean of three replicates ± SDs.

**FIGURE 3:**
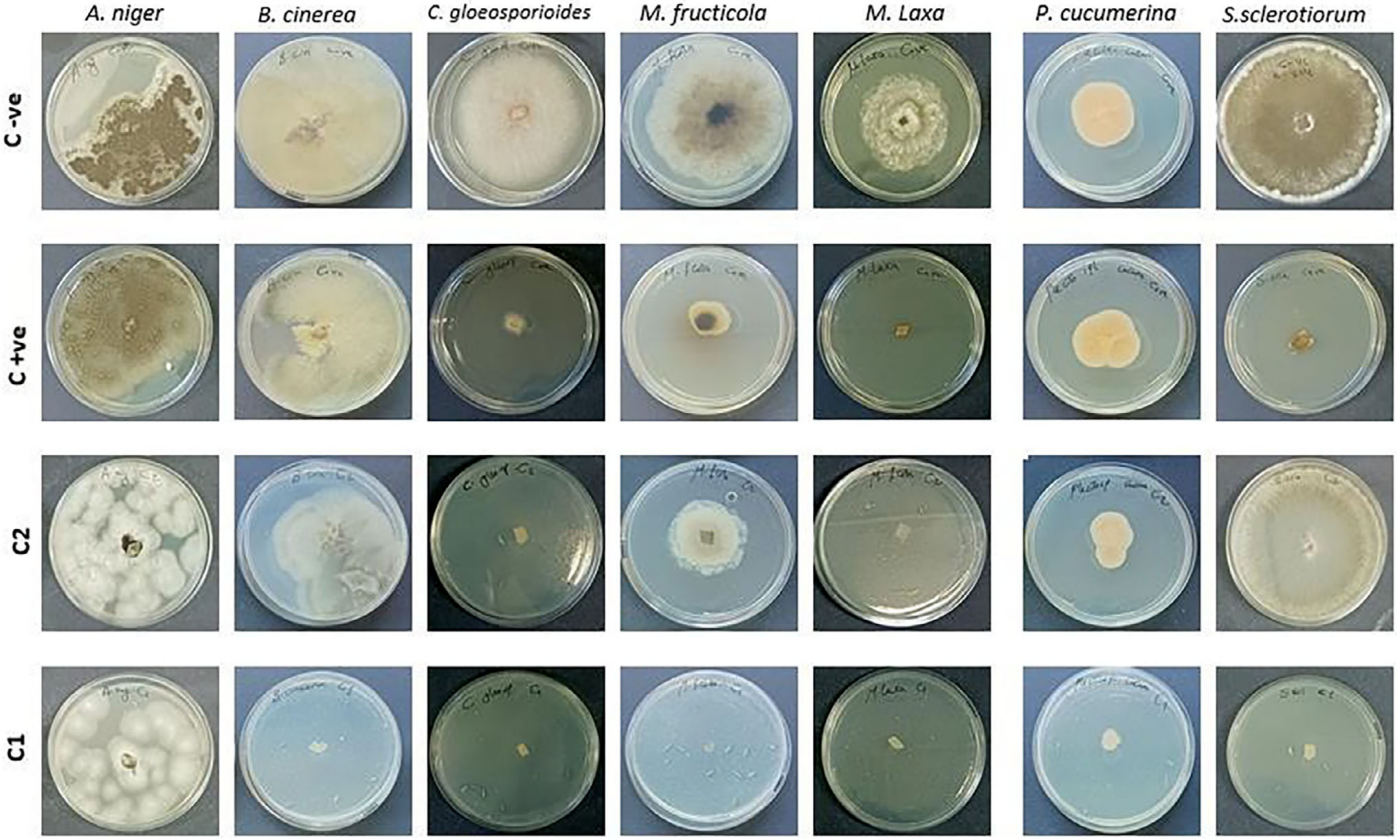
The antifungal activity of *Cinnamomum camphora* EO. Where C−ve is the negative control, C+ve is a mixture of azoxystrobin and cycloheximide, C1: 10 000 ppm, C2: 7000 ppm.

The antifungal study indicates that *C. camphora* EO showed significant antifungal activity against phytopathogenic fungi in a dose‐dependent manner (Figures 2 and 3). This EO showed greater efficacy than the standard used (azoxystrobin/cycloheximide) against all strains tested except for *A. niger*. Several studies have shown that a specific fungus can react differently to one EO compared to another, which makes fungi sensitive to some EOs and resistant to others [58, 59, 60]. The sample caused 100% inhibition of mycelial growth of *M. laxa, M. fructicola, S. sclerotiorum, C. gloeosporioides*, and *B. cinerea* at the tested concentration 10 000 ppm, whereas 80% of *P. cucumerina* at the same concentration. On the other hand, *A. niger* showed lower sensitivity to the tested EO (17.8%), with inhibition of the production of spores at the higher tested concentration 10 000 ppm. At the concentration of 7000 ppm, *M. laxa*, *M. fructicola*, *C. gloeosporioides*, and *P. cucumerina* were inhibited at more than 50%, in contrast to *S. sclerotiorum, A. niger*, and *B. cinerea*, which were inhibited at 4.2%, 5.4%, and 29.4%, respectively (Table 3).

**TABLE 3:** Antifungal activity of *Cinnamomum camphora* essential oil (EO).

Mycelium inhibition (%)			
	10 000 ppm	7000 ppm	C+ve
*Monilinia laxa*	100	90.1	100
*Monilinia fructicola*	100	56.5	72.35
*Sclerotinia sclerotiorum*	100	4.2	92.81
*Colletotrichum gloeosporioides*	100	76.1	76.72
*Aspergillus niger*	17.8	5.4	3.57
*Plectosphaerella cucumerina*	80.0	51.3	26.95
*Botrytis cinerea*	100	29.4	13.5

*Note*: Where C+ve is a mixture of azoxystrobin and cycloheximide.

Several studies have reported the antifungal potential of *C. camphora* EO, supporting its use as a preservative for fruits and vegetables, a powerful alternative to chemical fungicides [61, 62]. To our knowledge, this is the first study of antifungal activity of *C. camphora* leaves EO against *M. laxa, M. fructicola, S. sclerotiorum*, and *P. cucumerina*. The antifungal activity of *C. camphora* EO showed significant antifungal activity against phytopathogenic fungi in comparison to positive control. According to the results obtained by Marei et al. [63], limonene and eucalyptol showed promising antifungal activity against *A. niger* and other plant pathogenic fungi. Camphor EO was the most effective EO against *A. niger*, and that camphor oil inhibited 97.4% of the live spores for *A. niger* [64]. Other studies reported that *C. camphora* EO has antimicrobial activity against *A. alternata*, *Curvularia lunata*, *Candida* sp., *Bacillus cereus*, and *Staphylococus aureus* [65, 66, 67]. Pitarokili et al. [68] tested the antifungal activity of *Salvia fruticosa* EO against some phytopathogenic fungi, and they found that the inhibition of *S. sclerotiorum* mycelium growth was accompanied by a decrease in sclerotia production. They attributed this activity to the synergy between the two dominant terpenes in the EO (1,8‐cineole and camphor).

The antifungal activity may be due to synergy between EO components, because camphor, limonene, α‐pinene, β‐pinene, and 1,8‐cineole had greater antifungal activity when used together than when each constituent was used alone [65, 69, 70]. EOs are rich in terpenes, which are characterized by their powerful antimicrobial activity [71]. This activity can be attributed to their lipophilic nature and low molecular weight, which enable them to disrupt the cell membrane, cause cell death, or inhibit the sporulation and germination of fungi [72].

### 2.6 Minimum Inhibitory Concentration (MIC) of Antifungal Activity

**FIGURE 4:**
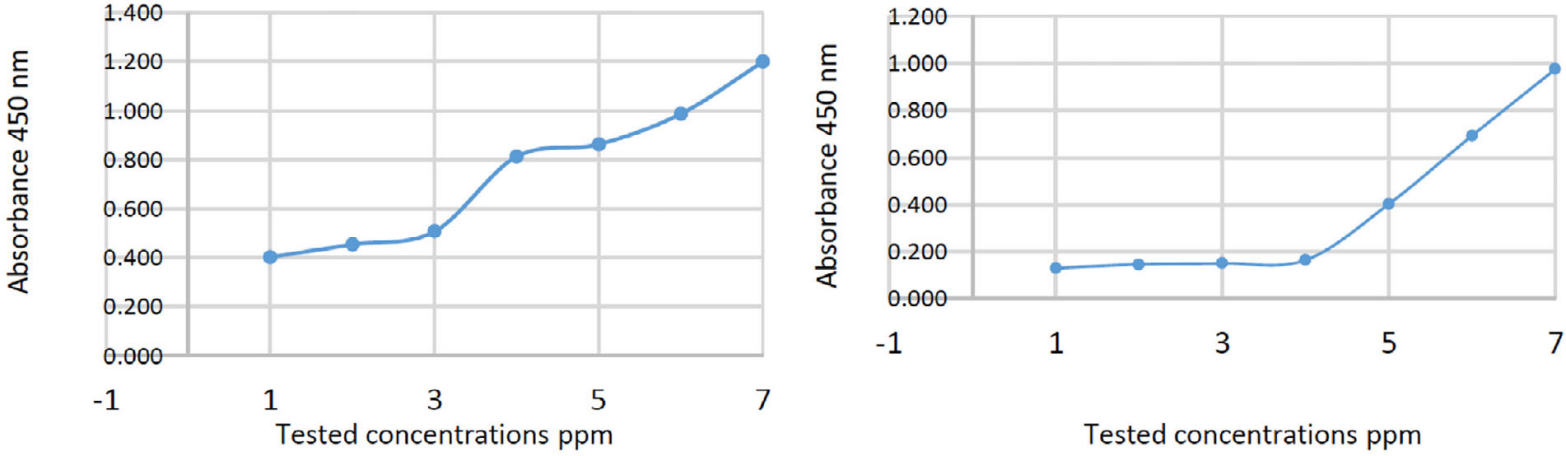
MIC values of fungal inhibition *Monilinia laxa* (right) and *Colletotrichum gloeosporioides* (left).

The MICs of *C. camphora* EO against *M. laxa* and *C. gloeosporioides* were 7000 and 8000 ppm, respectively (Table 4 and Figure 4). The MIC values obtained were higher than that obtained by Yan et al. [73], who tested the antifungal activity of *C. camphora* EO against *Trichoderma viride, A. niger*, and *Penicillium citrinum*, and the MIC was 500 µL/L for all three fungi, which indicates higher activity than that obtained by our EO. On the other hand, Oliveira Filho et al. [21] obtained lower antifungal activity of *C. camphora* EO compared to other EOs tested with 750 < MIC ≤ 1000 µL/L against *Rhizopus stolonifer*, but this value is still better than the MIC value obtained in our study.

**TABLE 4:** Minimum inhibitory concentrations (MIC) of studied *Cinnamomum camphora* essential oil (EO) against *Monilinia laxa* and *Colletotrichum gloeosporioides*.

Absorbance at 450 nm								
	C1	C2	C3	C4	C5	C6	C−ve	MIC ppm
*M. laxa*	0.130	0.146	0.150	0.164	0.402	0.692	0.975	7000
*C. gloeosporioides*	0.402	0.453	0.507	0.812	0.862	0.987	1.200	8000

*Note*: Where C1: 10 000, C2: 9000, C3: 8000, C4: 7000, C5: 6000, C6: 5000 (ppm), and C−ve: negative control plates containing only PDB.

## 3 Conclusion

The current study comprehensively demonstrates the promising bioactivity of *C. camphora* EO, supported by its complex chemical composition rich in oxygenated monoterpenes such as camphor, α‐pinene, and 1,8‐cineole. FTIR analysis confirmed the presence of key functional groups characteristic of these compounds. The EO exhibited moderate antioxidant potential, with appreciable ferric‐reducing capacity, highlighting its potential application as a natural antioxidant in the food and pharmaceutical industries. Although its inhibitory effects on AChE, α‐amylase, and α‐glucosidase were relatively modest compared to standard drugs, the EO still provides a promising foundation for developing natural enzyme inhibitors with reduced side effects. Most notably, the EO demonstrated potent antifungal activity against a range of economically significant phytopathogenic fungi, achieving complete inhibition of several species at high concentrations. These findings underscore its strong potential as an eco‐friendly alternative to synthetic fungicides, aligning with global efforts to reduce chemical pesticide usage and environmental contamination. The synergistic effects among the EO's terpenoid constituents likely contribute to its broad‐spectrum antimicrobial efficacy. Overall, *C. camphora* EO emerges as a valuable candidate for integrated pest management, as well as a bioactive agent in pharmaceutical and nutraceutical formulations. Future studies should focus on optimizing extraction methods, evaluating the EO's in vivo efficacy, and elucidating the molecular mechanisms underlying its bioactivities to pave the way for practical applications in sustainable agriculture and health care.

## 4 Experimental Section

### 4.1 Isolation and Chemical Investigation

The studied EO has been extracted from leaves of *C. camphora* plant located at the Botanical Extension Center, University of August 20, 1955, Skikda, North‐east Algeria. Sampling was carried out in March 2024, and the plant was identified by Dr. Sakhraoui Nora, Department of Ecology and Environment, University of August 20, 1955, Skikda. The voucher specimen of the plant (PPL 01/2023) was deposited in the herbarium of Plant Physiology Laboratory, Higher Normal School of Technological Education of Skikda, Algeria. The leaves were carefully separated from their peduncles and cleaned and left to dry for 15 days in darkness. After that, 100 g of the leaves was subjected to hydrodistillation for 3 h using a Clevenger apparatus. The obtained EO was stored in a refrigerator at 4°C until used. The chemical analysis of volatile oils was carried out by GC–MS, as described by [30].

### 4.2 Fourier‐Transform Infrared Spectroscopy

The FTIR analysis was applied to the studied EO sample to confirm the presence of functional groups. The experiment was performed by using a SHIMADZU FTIR‐8000 spectrometer (Tokyo, Japan) apparatus equipped with a single‐reflection ATR accessory and IR Pilot software.

A small drop of the EO was placed on the ATR crystal, and the spectrum was acquired in the range of frequency of 4000–600 cm^−1^, with a wavenumber resolution of 4 cm^−1^. The measurement was performed in duplicate, and the recorded IR spectrum represents the average of 20 scans.

### 4.3 Antioxidant Activity

#### 4.3.1 DPPH Assay

Antiradical activity of the studied EO was evaluated using DPPH assay following the methodological procedures of the basic principles of Brand‐Williams et al. [74]. This method was used for determining the antiradical activity of a compound based on the use of the stable free radical DPPH. The mechanism is based on the ability of tested compounds to reduce the DPPH radical (deep violet) into a neutral stable molecule (pale yellow). A stock radical solution of DPPH was prepared by dissolving 20 mg of DPPH in 15 mL ethanol.

This method was used for determining the antiradical activity of EO dissolved in DMSO at 125, 250, 500, and 1000 µg/mL. Fifty µL of each concentration was mixed with DPPH (1:20, v/v) and incubated in darkness for 30 min at room temperature (25°C). All samples were centrifuged at 7155 × *g* for 5 min, and the absorbance was measured at 515 nm on a UV/VIS spectrophotometer (LKB Biochrom 4050 Ultrospec II, Cambridge, England), whereas ethanol was used as a reference sample. Ascorbic acid was used as a reference standard. The solutions were prepared fresh for the analysis, and all determinations were carried out in triplicate and the evaluation of DPPH % using the following equation:

$$\mathrm{DPPH}\;\%=\frac{\mathrm{Abs}.\;C\;\left(\lambda \;515\right)-\mathrm{Abs}.\;S\;\left(\lambda \;515\right)}{\mathrm{Abs}.\;C\;\left(\lambda \;515\right)}\times \;100$$

where Abs. *C* is the absorbance of control with no radical scavenger, and Abs. *S* is the absorbance of remaining DPPH in the presence of a scavenger.

#### 4.3.2 ABTS^•+^ Free Radical Scavenging Activity

The ABTS^•+^ radical assay was carried out according to the protocol reported by Ud‐Daula et al. [75] with minor modifications. The mixture of ABTS (7 mM) and potassium persulfate (2.45 mM) solutions was incubated in the dark at 25°C for 16 h to generate the ABTS radical (ABTS⁺) prior to use. The ABTS+ solution was diluted with distilled water to an OD of 0.800 at 734 nm. Ten µL of different concentrations dissolved in methanol (0.1–1 mg/mL) and 190 µL of ABTS were placed in the wells for analysis. Ten µL of sodium phosphate buffer and 190 µL of distilled water were added to the wells for the control. The results were expressed as IC_50_ values, which are the amount of EOs necessary to reduce the radical ABTS activity by 50%. Trolox (6‐hydroxy‐2,5,7,8‐tetramethylchroman‐2‐carboxylic acid) was used as the drug reference.

### 4.4 FRAP Assay

The FRAP assay was carried out according to the method described in the literature [76]. Briefly, a solution consisting of a 10:1:1 ratio of 23 mM acetate buffer (pH 3.6), 10 mM of tripyridyl triazine (TPTZ) in HCl (40 mM), and FeCl_3_ (20 mM), respectively, was prepared as the FRAP reagent. The assay was performed in 96‐well microplate. In each well, 264 µL of FRAP reagent and 8 µL of EO dissolved in methanol at different concentrations (0.1–1 mg/mL) were placed. The reaction mixture was incubated at 37°C for 30 min in dark conditions. Absorbance was read at 593 nm using a Thermo Scientific Multiskan GO spectrophotometer (Thermo Fischer Scientific, Vantaa, Finland). The absorbance of the blank (FRAP reagent) was subtracted from all absorbances of the sample to determine the FRAP value for each sample. Trolox was used as the drug reference.

### 4.5 Anti‐Enzymatic Activity

#### 4.5.1 Cholinesterases Inhibition

The AChE inhibitory activity for the studied EO was carried out using Ellman's colorimetric method [77] with some modifications. Ten µL of different concentrations of EOs dissolved in methanol (50, 25, 12.5, 6.25 mg/mL) were previously prepared, then 415 µL of a solution of Tris–HCl (0.1 M, pH 8) and 25 µL of ACHE were added to each concentration, and the mixture was placed in the incubator (15 min, 37°C). Seventy‐five uL of acetylthiocholine iodide (AChI) and 475 µL of 5,5‐dithio‐bis‐2‐nitrobenzoic acid (DTNB) were added. The reactants were mixed in a 96‐well microplate. The mixture was pre‐incubated at 37°C for 30 min. Absorbance was read at 412 nm in a spectrophotometer (Thermo Fischer Scientific, Vantaa, Finland). Galantamine was the reference drug. The inhibition of the enzymatic activity of the enzymes studied was calculated as a percentage using the following formula:

$$\mathrm{Inhibition}\left(\%\right)=[(\mathrm{AC}-\mathrm{AE})/\mathrm{AC}]\times 100$$

where AC represents negative control absorbance; and AE represents enzyme solution absorbance after contact with EOs. The IC_50_, which represents the EO concentration that causes 50% inhibition, was derived by plotting the percentage inhibition versus sample concentrations. The experiments were carried out in triplicate.

### 4.6 α‐Amylase Inhibition Assay

In this study, the assessment of amylase activity was carried out according to the method of Bernfeld [78], with some modifications. One hundred µL of different concentrations of EO solubilized in methanol (10, 5, 2.5, 1.25 mg/mL), along with 200 µL of phosphate buffer (20 mM with pH = 6.9) and 100 µL of aqueous alpha‐amylase solution (1 U/mL), were mixed and then placed in a tube for 10 min at 37°C. Then, 180 µL of a starch solution (1% w/v) was added, and the reaction continued after incubation (at 37°C for 10 min). A volume of 180 µL of dinitrosalicylic acid reagent (ADNS) was added, and the mixture was incubated (at 100°C for 10 min). The absorbance was read at 540 nm with a Thermo Scientific Multiskan GO (Thermo Fischer Scientific, Vantaa, Finland). The experiments were carried out in triplicate. The inhibition of the enzymatic activity of α‐amylase was calculated as a percentage using the same formula used for cholinesterases.

### 4.7 α‐Glucosidase Inhibition Assay

In this test, 10 µL of different concentrations of EO solubilized in methanol (10, 5, 2.5, 1.25 mg/mL) and 150 µL of phosphate buffer (0.1 M, pH 7.0), together with 15 µL of 1 U/mL α‐glucosidase enzyme solution, were placed in a 96‐well plate. The plate was incubated at 37°C for 5 min. Then, 75 µL of 2.0 mM 4‐nitrophenyl‐d‐glucopyranoside (pNPG) was added, and the plate was incubated again for 10 min at 37°C. The absorbance was read at 405 nm using a Thermo Scientific Multiskan GO spectrophotometer (Thermo Fischer Scientific, Vantaa, Finland). Acarbose was used as a positive control. The experiments were carried out in triplicate. The inhibition of the enzymatic activity of α‐glucosidase was calculated as a percentage using the same formula used for cholinesterases and α‐amylase [79].

### 4.8 Antifungal Activity

The tested microscopic fungi were obtained from the fungal collection of the Department of Agricultural, Forestry, Food and Environmental Sciences (DAFE), University of Basilicata, Potenza (Italy), and were sub‐cultured on PDA nutrient media for 96 h at 24°C. The tested fungi were previously identified by morphological and molecular methods. The antifungal activity of the studied *C. camphora* EO was tested against seven phytopathogenic fungi: *M. laxa* (Aderh. & Ruhland) Honey, *M. fructicola* (G. Winter) Honey, *S. sclerotiorum* (Lib.) de Bary, *C. gloeosporioides* (Penz.) Penz. & Sacc., *A. niger* Tiegh, *B. cinerea* Pers, and *P. cucumerina* (Lindf.) W. Gams. The studied EO was used at 7000 and 10 000 ppm incorporated into PDA as reported by Gruľová et al. [80]. Plates containing only PDA were considered the negative control, whereas the positive control was a mixture of azoxystrobin/cycloheximide (1:1; v/v) used at a final concentration of 0.8 µg/mL. All plates were left overnight until complete dryness, and then a 0.5 mm of agar disk of each tested fungus was inoculated in the center of the plates and incubated at 22°C ± 2°C for 96 h. The antifungal activity was determined by measuring the diameter of the fungal mycelium (mm), and the percentage of mycelium growth inhibition (MGI %) was calculated using the following equation:

$$\mathrm{MGI}\;\left(\%\right)=\frac{\mathrm{DM}\;\;\mathrm{control}\;\mathrm{plates}-\mathrm{DM}\;\;\mathrm{treated}\;\mathrm{plates}}{\mathrm{DM}\;\;\mathrm{control}\;\mathrm{plates}}\times 100$$

where MGI % is the mycelium growth inhibition percentage; DM is the diameter of mycelium (mm).

### 4.9 Determination of the Antifungal MIC (96‐Well Microplate Method)

The antifungal MIC of *C. camphora* EO was evaluated against *M. laxa* and *C. gloeosporioides* on a *96*‐well microplate [81]. Briefly, different concentrations of EO at 10 000, 9000, 8000, 7000, 6000, and 5000 ppm were inoculated with each single fungal suspension and Potato Dextrose Broth (PDB), then incubated for 48 h at 22°C. The absorbance was read using a microplate reader instrument (DAS s.r.l., Rome, Italy) at 450 nm.

### 4.10 Statistical Analysis

The obtained results were statistically analyzed, applying one‐way ANOVA using the Statistical Package for the Social Sciences (SPSS) version 13.0 (Prentice Hall: Chicago, IL, USA, 2004). The Tukey B post hoc multiple comparison test was used to evaluate the significance level with a probability of *p *< 0.05.

## Author Contributions

**Maroua Cheribot Cherif**: formal analysis, investigation, writing original draft. **Hicham Boughendjioua**: conceptualization, supervision, validation. **Lucia Caputo**: formal analysis, data curation, investigation. **Ippolito Camele**: conceptualization, supervision, validation, investigation, writing – review and editing. **Tarek Tahraoui**: conceptualization, investigation, visualization. **Vincenzo De Feo**: investigation, supervision, validation, writing – review and editing. **Hazem S. Elshafie**: conceptualization, formal analysis, data curation, validation, writing – review and editing.

## Disclosure

This manuscript reflects only the authors’ views and opinions; neither the European Union nor the European Commission can be considered responsible for them.

## Consent

The authors have nothing to report.

## Conflicts of Interest

The authors declare no conflicts of interest.

## Data Availability

The data that support the findings of this study are available from the corresponding author upon reasonable request.
